# MRCP Combined With CT Promotes the Differentiation of Benign and Malignant Distal Bile Duct Strictures

**DOI:** 10.3389/fonc.2021.683869

**Published:** 2021-09-14

**Authors:** Guang-xian Wang, Xiao-dong Ge, Dong Zhang, Hai-ling Chen, Qi-chuan Zhang, Li Wen

**Affiliations:** ^1^Department of Radiology, Xinqiao Hospital, Chongqing, China; ^2^Department of Radiology, Banan People’s Hospital of Chongqing, Chongqing, China; ^3^Department of Pathology, Xinqiao Hospital, Chongqing, China

**Keywords:** distal biliary strictures, MRCP, CT, imaging findings, risk factors

## Abstract

**Objective:**

To determine whether contrast-enhanced computed tomography (CT) can promote the identification of malignant and benign distal biliary strictures (DBSs) compared to the use of magnetic resonance cholangiopancreatography (MRCP) alone and to identify imaging findings of malignant DBSs.

**Materials and Methods:**

A total of 168 consecutive patients with confirmed DBSs were reviewed. MRCP alone and MRCP combined with CT images were blindly analyzed by two radiologists (e.g., stricture pattern, margins), and malignant or benign DBSs were identified based on surgical findings, endoscopy findings, or follow-up. The diagnostic accuracy of the two reviewers using MRCP alone and MRCP combined with CT were evaluated. MRCP and CT features of malignant and benign DBSs were compared using multiple logistic regression analysis to identify independent malignant risk factors.

**Results:**

MRCP combined with CT examination could improve the diagnostic accuracy, which increased from 70.2% to 81.5% in Doctor A and from 85.1% to 89.3% in Doctor B. The multiple logistic regression model revealed that stricture length [odds ratio (OR) 1.070, *P*=0.016], angle of the DBS (OR 1.061, *P*<0.001), double duct sign (OR 4.312, *P*=0.003) and low density in the arterial phase (OR 0.319, *P*=0.018) were associated with malignant DBS. A scoring model incorporating these four factors was established; at a threshold value of 1.75, and the sensitivity and specificity for the detection of malignant DBSs were 73.5 and 85.9%, respectively.

**Conclusions:**

Compared to the use of MRCP alone, MRCP combined with contrast-enhanced CT can improve the accuracy of DBS diagnosis. The scoring model accurately predicts malignant DBSs and helps make treatment decisions.

## Introduction

It remains difficult to differentiate between benign and malignant biliary strictures (BSs) (1–3). BSs result from various etiologies, and although the majority are malignant, up to 30% of BSs are benign (2, 4). In addition, 15-24% of suspected malignant strictures are determined to be benign after surgical resection (1, 5). The reason for this dilemma is that a focal malignant stricture without an identifiable mass sometimes mimics a benign lesion (6, 7). Occasionally, a benign stricture manifests as a focal area of wall thickening and mimics a malignant lesion (7, 8), and surgical resection may be performed. However, unnecessary surgery may delay appropriate treatment and lead to deterioration of patient condition (9). In clinical practice, early and accurate preoperative diagnosis of the cause of a BS is important to increase the likelihood of complete resection and to avoid unnecessary surgery.

Relative to proximal bile duct disease, distal biliary strictures (DBSs) are more complicated diagnose because the anatomy is close to the pancreas and duodenum (10). Endoscopic retrograde cholangiopancreatography (ERCP) and endoscopic ultrasonography (EUS) are the most widely used endoscopic diagnostic modalities in suspected DBSs (4); however, these techniques are invasive and operator dependent (11–13) and may lead to complications such as seeding metastasis (4) or pancreatitis (14). In addition, the combination of biliary brushing and intraductal biopsy has a sensitivity of only 60%–70% (15). Noninvasive imaging techniques, including ultrasound (US), computed tomography (CT) and magnetic resonance cholangiopancreatography (MRCP), are the common initial examination methods used for suspected DBSs prior to the use of these more invasive techniques. Transabdominal US has been considered the initial imaging test because of its accessibility, speed, ease of performance and low cost (1, 16, 17). However, US has limited ability to detect DBSs (1–4, 16) because the distal common bile duct is difficult to visualize, and bowel gas shadows may obscure the details. MRCP has become the preferred imaging technique for the evaluation of DBSs because of its high spatial resolution of the biliary tree; moreover, it is noninvasive and does not expose the patient to ionizing radiation (1, 7, 11, 16). Previous MRCP studies have confirmed the role of MRCP in the differentiation of malignant and benign causes of BSs (7, 16, 18, 19). However, an evaluation of the wall of the biliary duct and the extension of the malignancy based on MRCP alone is not accurate enough (6, 11).

Given advances in multidetector-row helical CT (MDCT) technology and its high imaging resolution as well as the availability of thin sections, the use of CT is becoming more common (20, 21). In our clinical practice, patients with DBSs usually undergo contrast-enhanced CT and MRCP. Thus, the purpose of this study was twofold: a) to compare the diagnostic accuracy of MRCP combined with contrast-enhanced CT and MRCP in differentiating malignant DBSs from benign DBSs and b) to determine the imaging findings of malignant DBSs.

## Materials and Methods

### Patients

The ethics office of the study institution approved this retrospective study, waiving the requirement for informed consent. The terms “MRCP”, “CT”, and “distal biliary strictures” were searched from the picture archiving and communication system (PACS) at our hospital among records from August 2011 through April 2020. A total of 2437 patients had demonstrable DBSs. Patients with cholangiolithiasis (*n* = 1280), exophytic or polypoid cholangiocarcinoma of the DBSs (*n* = 13), diseases of the pancreas (e.g., pancreatic cancer, acute or chronic pancreatitis) (*n* = 658), duodenal disease (e.g., duodenal cancer) (*n* = 96), congenital diseases (e.g., biliary atresia and Alagille syndrome) (*n* =4) or lymph node compression (*n* = 67) were excluded. Patients who underwent biliary interventional procedures (e.g., biliary stent insertion and endoscopic biliary drainage) before the MRCP and CT examination (*n* = 94), patients who underwent MRCP and CT examination 1 month prior to surgery or endoscopy (*n* = 11), patients whose images were of poor quality for analysis (*n* = 29), and patients who were diagnosed with DBSs with no histological proof or follow-up examination were also excluded (*n* = 17). Liver transplant patients were excluded from the study.

Finally, 168 patients with DBSs were enrolled in this study (**Table 1**). This total included 83 patients with malignant DBSs (age range 17-86 years) and 85 patients (age range 16-85 years) with benign DBSs. Patient clinical characteristics were not associated with different prevalence of malignant DBSs. In patients with malignant DBSs (cholangiocarcinoma), the diagnosis was based on histopathological findings after the pancreatoduodenectomy surgery (*n* = 71), ERCP biopsy (*n* = 11), or EUS biopsy (*n* = 1). Patients with benign DBSs diagnosed with inflammation after pancreaticoduodenectomy (n = 17), ERCP biopsy (n = 41), or EUS biopsy (n = 2), and the remaining 25 patients without treated were followed with CT or MRCP, showing no remarkable changes on imaging studies during a follow-up period of more than 12 months.

**Table 1:** The clinical characteristics of patients with DBSs.

Clinical data	Malignant DBSs (n = 83)	Benign DBSs (n = 85)	*P*
Sex			0.122
Female	39 (47.0%)	51 (60.0%)	
Male	44 (53.0%)	34 (40.0%)	
Age (years)	62.28 ± 11.56	59.54 ± 13.02	0.154
CA19-9 (U/mL)	188.67 ± 305.66	129.87 ± 256.75	0.178
Direct bilirubin (μmol/L)	45.15 ± 57.22	30.47 ± 58.74	0.103
Total bilirubin (μmol/L)	84.98 ± 97.35	63.30 ± 94.30	0.145
ALT (U/L)	80.03 ± 80.60	90.02 ± 140.94	0.575
AST (U/L)	82.53 ± 118.74	76.90 ± 10.67	0.747

DBSs, distal biliary strictures; ALT, alanine aminotransferase; AST, aspartate aminotransferase.

### Image Protocols

All patients fasted for 8 hours before MRCP and CT examination. The time interval between MRCP and CT varied from 0 to 7 (1.53 ± 1.95) days.

MRCP was performed using a 1.5 T clinical MRI system (Signa, GE Medical System, Milwaukee, WI, USA) and phased-array surface coil. Conventional sequences, such as 2D axial T1-weighted images [repetition time (TR)/echo time (TE) 180-220/4.7 ms], 2D axial T2-weighted images (TR/TE 6000-10000/85 ms) and a coronal fast imaging employing steady-state acquisition (FIESTA) sequences (TR/TE 4.7/1.5 ms), were conducted with a slice thickness of 6 mm, intersection overlap of 1 mm, and field of view (FOV) of 34-38 mm. 3D-MRCP was performed using fast recovery fast spin echo (FRFSE) in the coronal plane. The parameters were as follows: TR/TE 4000-7500/730-1000 ms, slice thickness 1.6 mm, no gap between sections, matrix 288×256, and FOV 20-40 mm. Fat saturation was employed to suppress interference from the surrounding fat tissues. The T2-weighted images were obtained with free breathing, and the other images were obtained with the patients holding their breath to reduce the artifacts created by respiratory motion.

Generally, patients drank 800-1000 ml of water before CT examination to distend the gastrointestinal tract (unless the patient was on dietary restrictions). CT scans were performed with a 64-slice LightSpeed VCT machine (GE Medical System, Milwaukee, WI, USA) or a dual-source scanner (Flash, Siemens Medical Solutions, Erlangen, Germany). The following parameters were used: 120 kVp, 200-280 mA s, collimation 64 × 0.625 mm or 128 × 0.6 mm, matrix 512 × 512, pitch 0.6-1.0, section thickness and section distance 5 mm. A total of 80-100 ml of nonionic contrast medium (Visipaque 320; GE Healthcare) was intravenously administered at a rate of 3 to 3.5 ml/s *via* an automatic injector. After contrast medium injection, the arterial phase (25-30 s), portal venous phase (50-60 s) and delayed phase (120 s) were obtained. Subsequently, all images were thin-sliced at a thickness of 0.625 mm and transferred to PACS. Multiplanar reformations (MPRs) can be performed by PACS.

### Image Analysis

Given that the accuracy of the differentiation of malignant from benign causes of obstruction range widely, from 38 to 90% (22), the diagnosis of malignant or benign DBSs in our center depends mainly on experience. MRCP and CT images were analyzed by two abdominal radiologists; one (doctor A) with five years of experience, and the other (doctor B) had 20 years of experience in abdominal radiology. The two observers, blinded to the clinical data and pathological diagnosis analyzed the images independently. Observation and measurement were performed using PACS, and continuous data were calculated as average values, while any discrepancies in categorical data were re-evaluated by a third reader (20 years of experience in abdominal radiology) for subsequent statistical analyses.

**Figure 1:**
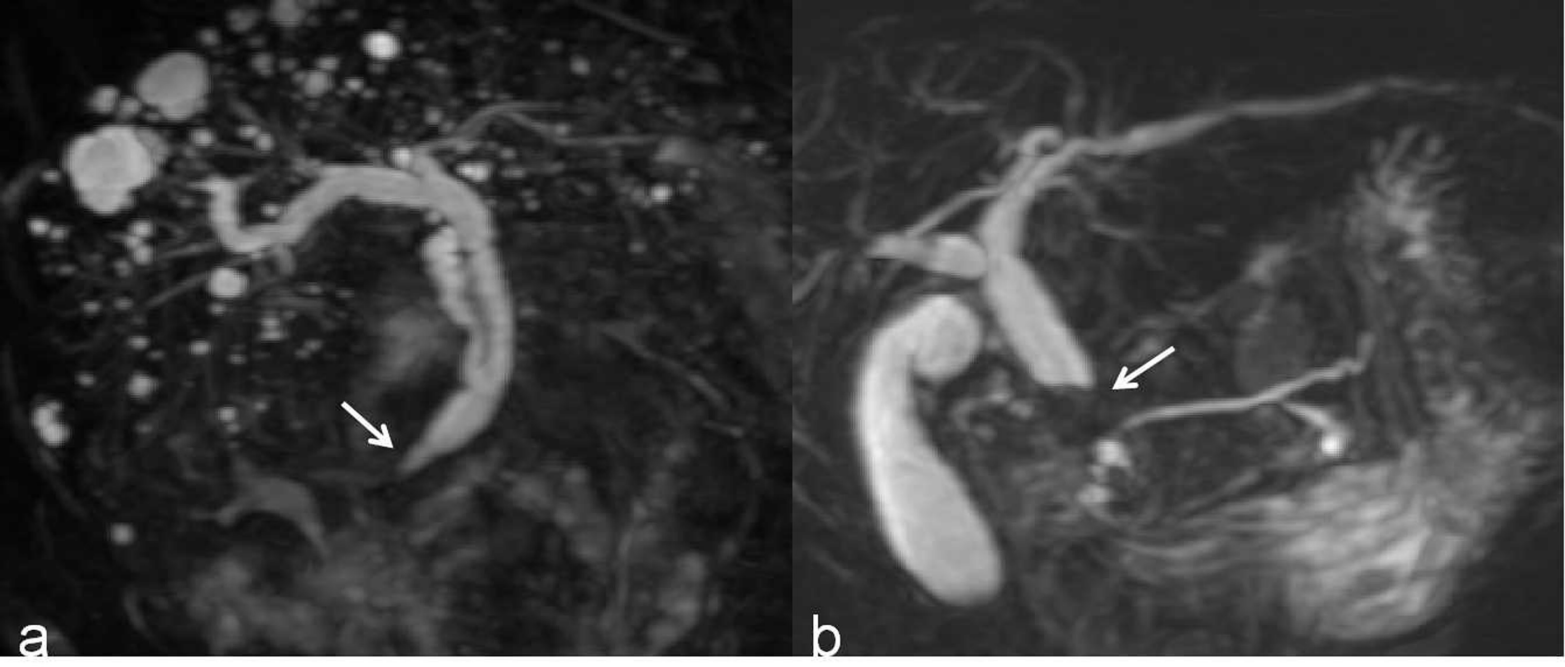
**(A)** A 3D MRCP image shows a DBS with gradual tapering, concentric and symmetric narrowing, acute angle of the narrowing end, smooth margins, and absence of the double duct sign (white arrow). **(B)** A 3D MRCP image shows a DBS with abrupt, eccentric and asymmetric narrowing, obtuse angle of the narrowing end, irregular margins, and the presence of the double duct sign (white arrow).

**Figure 2:**
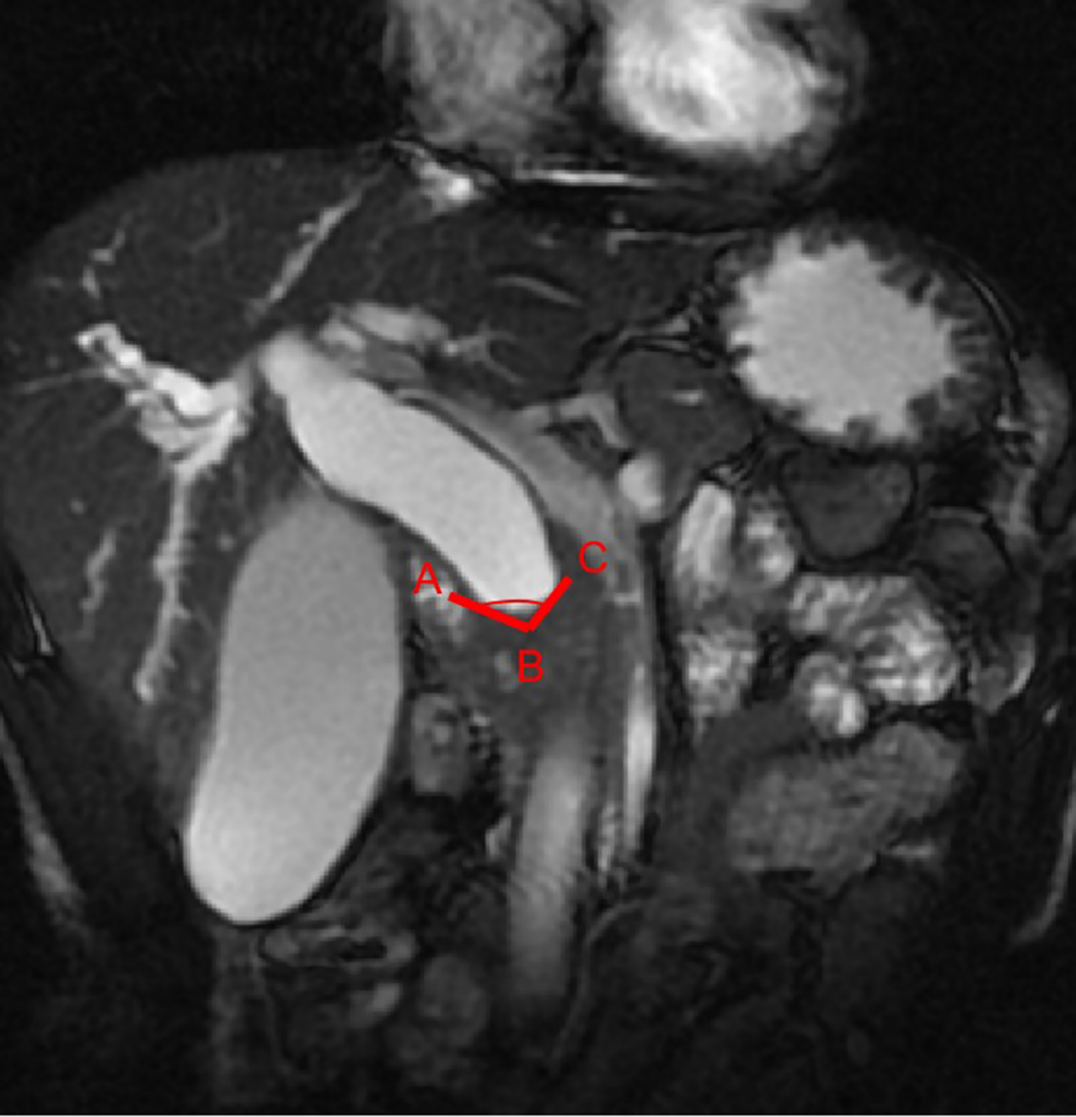
The stenosis angle (∠ABC) is defined as the angle between two intersecting lines (AB and BC) which passes through the midpoint of the proximal terminal of the stenosis (point B) and the crosspoints of the two walls of the common bile duct and the proximal terminal of the stenosis (point A and C) on coronal FIESTA image.

First, the two observers analyzed the MRCP features independently and identified malignant or benign DBSs. Dichotomous morphological variables included abrupt narrowing or gradual tapering, concentric or eccentric, asymmetric or symmetric narrowing, obtuse or acute angle of the narrowing end, irregular or smooth margins, and the presence or absence of the double duct sign (**Figure 1**). A main pancreatic duct diameter larger than 2 mm was considered dilatation (normal range ≤2 mm). In addition, the maximum length of the DBS, the maximum diameter of common bile duct dilatation and the angle of the DBS were also recorded. The angle of the DBS measurement is shown in **Figure 2**.

One month later, the same two observers analyzed the MRCP and CT images concurrently to identify malignant or benign DBSs again. The maximum wall thickness of the DBS was measured on contrast-enhanced CT. In addition, they also classified each lesion according to its appearance, such as enhancement patterns (rim or nonrim enhancement) and enhancement degree (low, iso-, or high). Rim enhancement was defined as the presence of an enhanced duct wall surrounding the lumen, and nonrim enhancement was defined as no enhancement or focal enhancement. The CT numbers were measured by means of same-size region of interest (ROI) cursors placed on wall portions of the DBS and on the adjacent liver parenchyma. The reviewers drew the ROI within three portions of the lesion and the liver parenchyma, after which the mean CT numbers were calculated. The enhancement degree of the DBS wall was compared with that of the liver parenchyma, and a difference of more than 10 Hounsfield units in mean CT number between the DBS wall and the liver parenchyma was considered meaningful (8).

### Statistical Analysis

Statistical analyses were performed using SPSS software (version 17.0; SPSS Chicago, IL, USA). A *P* value less than 0.05 was considered to be a statistically significant difference. The interobserver reliability of morphological parameters was calculated using Kappa consistence test. Continuous variables are expressed as the mean ± SD, and categorical variables are expressed as the number and percentage. Differences between the categorical variables were compared by using the *chi*-squared test. Continuous variables with a normal distribution were assessed by independent *t-*test; otherwise, the Mann-Whitney *U*-test was used for non-normally distributed data. When the *P* values for variables were less than 0.05, they were entered into multiple logistic regression to calculate the independent risk factors and the odds ratios (ORs) and 95% confidence intervals (CIs) for the likelihood of malignancy. Receiver operating characteristic (ROC) curve analysis was performed to determine the cutoff value at which the value of (sensitivity+specificity–1) reached its maximum.

## Results

The diagnostic accuracy of the two reviewers using MRCP alone and MRCP combined with CT is listed in **Table 2**. When using MRCP alone, for doctor A, the sensitivity, specificity, positive predictive value, negative predictive value, and diagnostic accuracy were 68.7% (57/83), 71.8% (61/85), 70.4% (57/81), 70.1% (61/87), and 70.2% [(57 + 61)/168], respectively. For doctor B, the sensitivity, specificity, positive predictive value, negative predictive value, and diagnostic accuracy were 90.4% (75/83), 80.0% (68/85), 81.5% (75/92), 89.5% (68/76), and 85.1% [75 + 68)/168], respectively. When combining with CT, for doctor A, 32 false lesions were corrected, but 13 correct cases were revised incorrectly. The sensitivity, specificity, positive predictive value, negative predictive value, and diagnostic accuracy were 84.3% (70/83), 78.8% (67/85), 79.5% (70/88), 83.8% (67/80), and 81.5% [(70 + 67)/168], respectively. For doctor B, 8 false lesions were corrected, and 1 correct case was revised incorrectly. The sensitivity, specificity, positive predictive value, negative predictive value, and diagnostic accuracy were 90.4% (75/83), 88.2% (75/85), 88.2% (75/85), 90.4% (75/83), and 89.3% [(75 + 75)/168], respectively.

**Table 2:** Diagnostic accuracy of the two reviewers using MRCP alone and MRCP combined with CT.

	MRCP alone	Results							
		Malignant	Benign	Total	SEN%	SPE%	PPV%	NPV%	DA%
Doctor A	Malignant	57	24	81	68.7	71.8	70.4	70.1	70.2
	Benign	26	61	87					
Doctor B	Malignant	75	17	92	90.4	80.0	81.5	89.5	85.1
	Benign	8	68	76					
Total		83	85	168					
	MRCP with CT								
Doctor A	Malignant	70	18	88	84.3	78.8	79.5	83.8	81.5
	Benign	13	67	80					
Doctor B	Malignant	75	10	85	90.4	88.2	88.2	90.4	89.3
	Benign	8	75	83					
Total		83	85	168					

SEN, sensitivity; SPE, specificity; PPV, positive predictive value; NPV, negative predictive value; DA, diagnostic accuracy.

There was moderate to excellent agreement between the two observers as indicated by the κ consistence test (κ values of 0.41–0.60 moderate agreement; κ values of 0.61–0.80 good agreement; κ values of 0.81–1.00 excellent agreement) (**Table 3**). The morphological characteristics of DBSs are listed in **Table 4**. Abrupt narrowing, eccentricity, obtuse angles, irregular margins, the presence of the double duct sign, stricture length, the angle of the DBS, the maximum diameter of common bile duct dilatation, wall thickness, enhancement patterns, and high density and low density in the arterial phase were associated with malignant DBSs. Then, these variables were entered into a forward conditional multiple logistic regression model (**Table 5**). The model showed that stricture length (OR 1.073), the angle of the DBS (OR 1.062), and the presence of the double duct sign (OR 4.162) were positively associated with malignant DBSs. In contrast, DBSs with low density in the arterial phase (OR 0.306) decreased the risk of malignancy. The threshold values of the stricture length and angle of the DBS were 9.5 mm and 84.1°, respectively, and the AUC values were 0.694 and 0.783, respectively.

**Table 3:** Inter-observer variability in morphological parameters.

Morphological parameters	κ coefficients
Abrupt narrowing or gradual tapering	0.521
Concentric or eccentric	0.433
Asymmetric or symmetric	0.669
Angle of the narrowing end	0.488
Margins	0.556
Present of double duct sign	0.902
Enhancement patterns	0.798
Enhancement degree	
Arterial phase	0.498
Portal venous phase	0.823
Equilibrium phase	0.688

**Table 4:** The morphological characteristics of DBSs.

Morphological parameters	Malignant DBSs (n = 83)	Benign DBSs (n = 85)	*P*
MRCP parameters			
Stricture pattern^1^			<0.001
Abrupt narrowing	67 (80.7%)	40 (47.1%)	
Gradual tapering	16 (19.3%)	45 (52.9%)	
Stricture pattern^2^			0.062
Concentric	53 (63.9%)	66 (77.6%)	
Eccentric	30 (36.1%)	19 (22.4%)	
Stricture pattern^3^			0.146
Asymmetric	34 (41.0%)	25 (29.4%)	
Symmetric	49 (59.0%)	60 (70.6%)	
Angle of the narrowing end			<0.001
Obtuse angle	62 (74.7%)	36 (42.4%)	
Abrupt angle	21 (25.3%)	49 (57.6%)	
Margins			0.002
Irregular	25 (30.1%)	9 (10.6%)	
Smooth	58 (69.9%)	76 (89.4%)	
Present of double duct sign	25 (30.1%)	9 (10.6%)	0.002
Stricture length (mm)	14.50 ± 7.01	10.60 ± 6.37	<0.001
Angle of the DBS (°)	96.50 ± 16.22	77.30 ± 18.44	<0.001
Maximum diameter of common bile duct (mm)	19.10 ± 6.02	14.50 ± 4.69	<0.001
CT parameters			
Wall thickness (mm)	2.87 ± 0.95	2.39 ± 0.65	<0.001
Enhancement patterns			<0.001
Rim enhancement	30 (36.1%)	54 (63.5%)	
Nonrim enhancement	53 (63.9%)	31 (89.4%)	
Enhancement degree			
Arterial phase			
High density	56 (67.5%)	38 (44.7%)	0.003
Isodensity	16 (19.3%)	20 (23.5%)	0.574
Low density	11 (13.2%)	27 (31.8%)	0.005
Portal venous phase			
High density	25 (30.1%)	16 (18.8%)	0.107
Isodensity	20 (24.1%)	22 (25.9%)	0.859
Low density	38 (45.8%)	47 (55.3%)	0.280
Equilibrium phase			
High density	22 (26.5%)	17 (20.0%)	0.363
Isodensity	31 (37.3%)	32 (37.6%)	1.000
Low density	30 (36.2%)	36 (42.4%)	0.433

DBSs, distal biliary strictures. ^1, 2, 3^ represent different patterns of stricture.

**Table 5:** Multiple logistic regression analysis for the prediction of malignant DBSs.

Variable	Odds ratio	*P*	95% CI	*β*
Stricture length	1.073	0.016	1.013–1.137	0.071
Angle of the DBS	1.062	<0.001	1.039–1.086	0.061
Double duct sign	4.162	0.003	1.598–10.836	1.426
Low density in arterial phase	0.306	0.018	0.116–0.813	–1.183

DBSs, distal biliary strictures; CI, confidence interval; β, partial regression coefficient.

**Figure 3:**
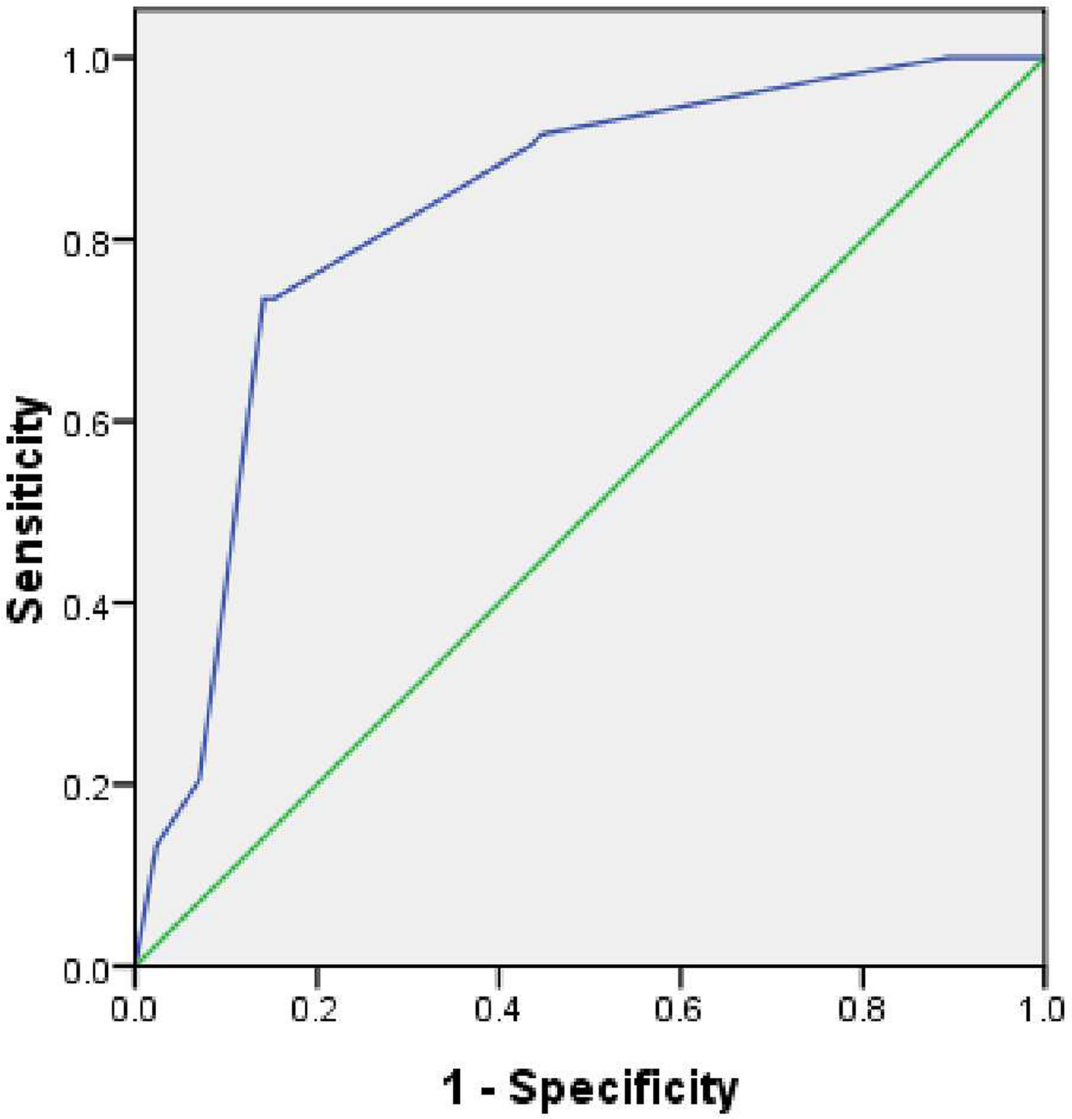
The area under the ROC curve for the predictive score was 0.828 (95% CI, 0.763-0.892). The cut-off point for the predictive score was 1.75, the sensitivity was 73.5%, and the specificity was 85.9%.

**Figure 4:**
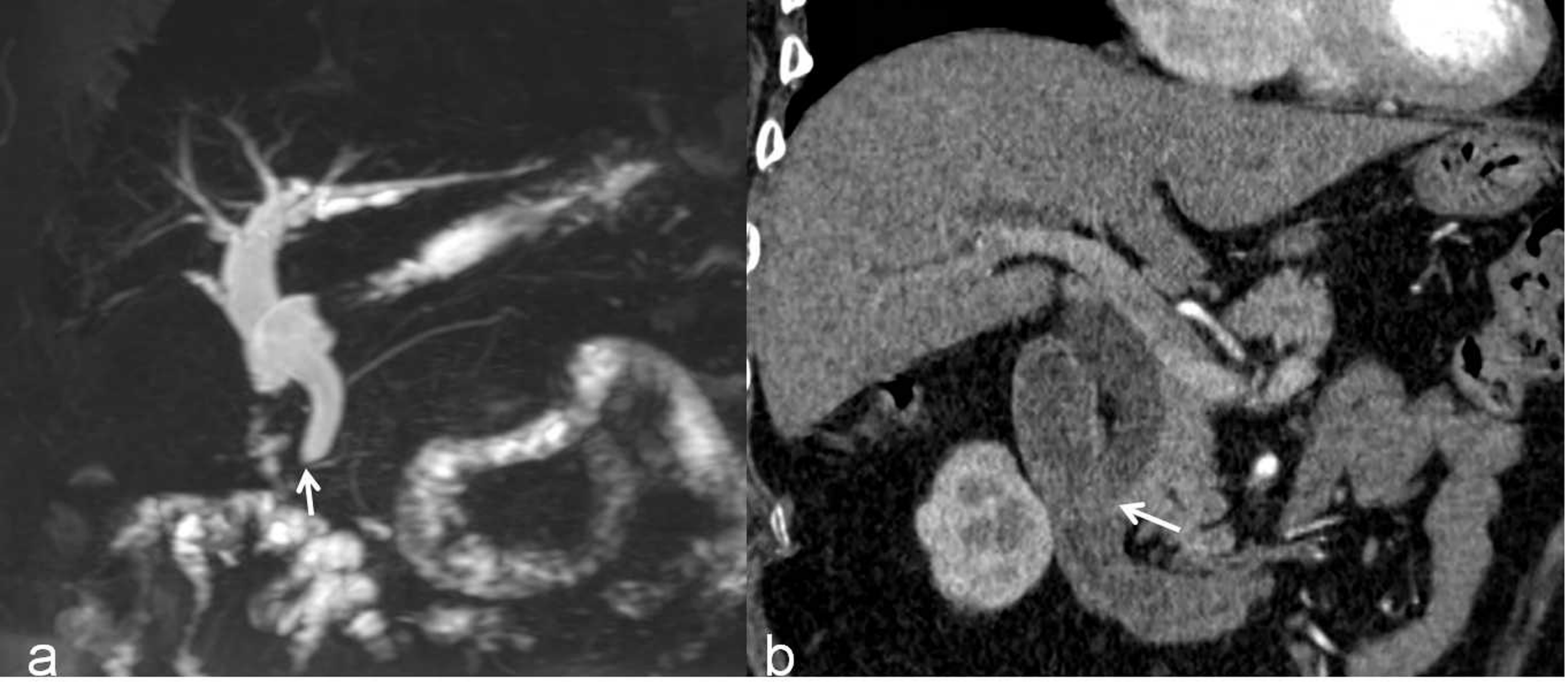
A benign DBS (score=0) in a 67-year-old woman who had a history of cholecystectomy. She did not require treatment but received imaging follow-up. **(A)** A 3D MRCP image shows a DBS with upstream bile duct dilatation. The stricture length is 4 mm, the angle of the DBS is 67.7°, and the double duct sign is not present. **(B)** A coronal CT MPR image demonstrates the isodensity of the wall in the arterial phase relative to the liver parenchyma (white arrow).

**Figure 5:**
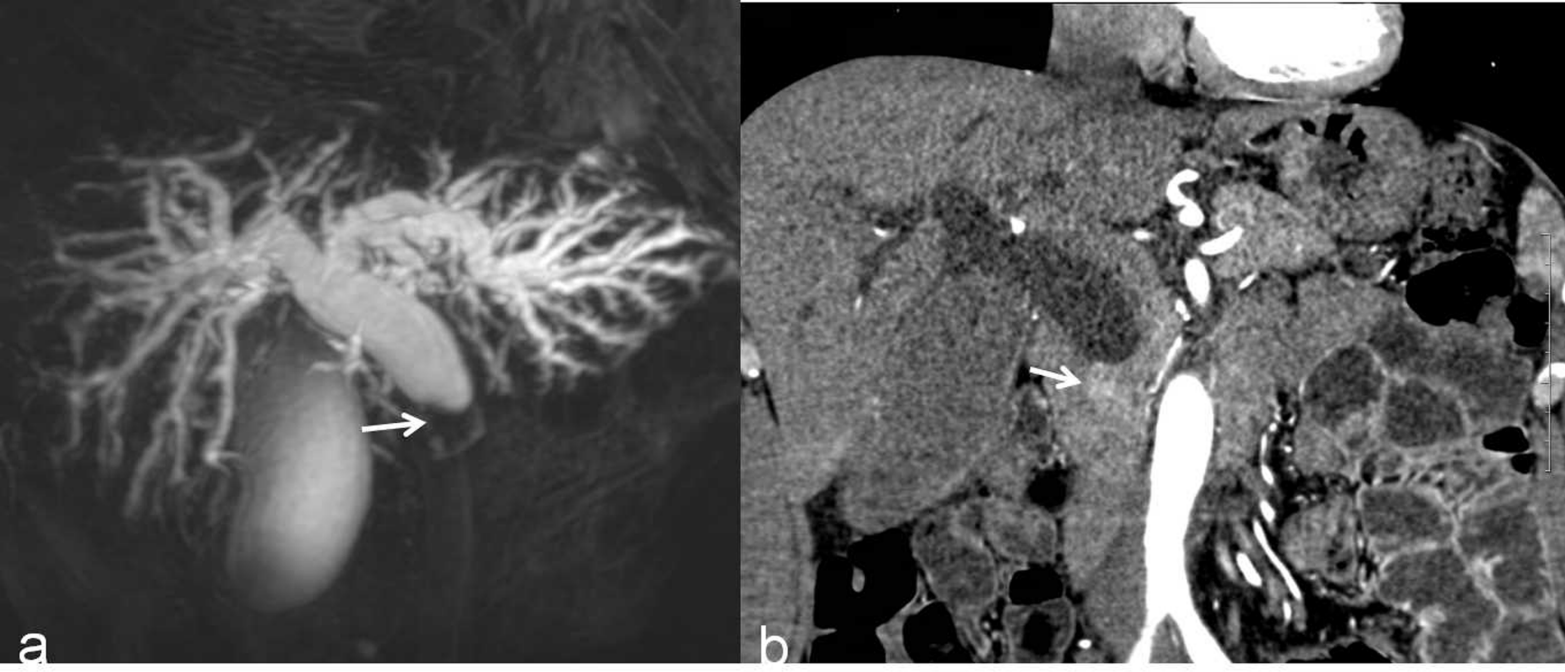
A malignant DBS (score=2) in a 59-year-old man confirmed the lesion as cholangiocarcinoma after surgery. **(A)** A 3D MRCP image shows abrupt, asymmetrical occlusion with irregular margins (white arrow). The length of the longest segment is 16 mm, the angle of the DBS is 100.4°, and the double duct sign is not present. **(B)** A coronal CT MPR image in the arterial phase shows the thickened wall with hyperenhancement relative to the liver parenchyma (white arrow).

According to the β coefficient, a predictive scoring model for malignant DBS risk was established, and points were assigned as follows. If the stricture length was ≥ 9.5 mm and the angle of the DBS was ≥ 84.1°, the score was 1; if the stricture length was < 9.5 mm and the angle of the DBS was < 84.1°, the score was 0. If the double duct sign was present, the score was 2.5; otherwise, the score was 0. If low density was present in the arterial phase, the score was –2; otherwise, the score was 0. Through ROC curve analysis, the AUC was 0.828; the optimal cutoff value of the predictive score was 1.75; the sensitivity and specificity for the detection of malignant DBSs were 73.5 and 85.9%, respectively; and the 95% CI was 0.763–0.892 (**Figure 3**). The incidence of malignant DBSs in the low-risk group (score < 1.75) and the high-risk group (score ≥ 1.75) was 23.2 (22/95) and 83.6% (61/73), respectively (**Table 6**) (**Figures 4**, **5**).

**Table 6:** Incidence of malignant DBSs based on the predictive scoring model.

Score	Total (n)	Malignant (n)	Risk	Incidence of malignancy
–2	9	0	low	23.2%
–1	14	2		
0	31	5		
0.5	2	1		
1	38	14		
1.5	1	0		
2	42	37	high	83.6%
2.5	8	7		
3.5	10	6		
4.5	13	11		

## Discussion

Patients with benign DBS can be treated by more conservative methods, such as ERCP drainage or follow-up; in contrast, for malignant DBS, early and complete resection is the primary treatment. However, despite improvements in imaging technologies, such as MRCP and endoscopic techniques, the differentiation of benign from malignant DBSs has remained a challenge for radiologists. Most of previous studies were based on CT, MRCP or MRCP combined with CE-MRI and focused on the extrahepatic bile duct or the full range of the bile duct, the risk factors for malignant BSs were not consistent (6–8, 11, 18, 20). Although MRI/MRCP is superior to CT in differentiating between malignant and benign BSs (1, 2, 16), the specificity of MRCP combined with CE-MRI still needs to be improved (15, 23), with a specificity of 70%–85% (15). Furthermore, CT is the preferred initial modality in differentiating between malignant and benign DBSs (24). To our knowledge, few studies have described the use of MRCP combined with CT for risk factors for malignant DBSs. In this study, we found that the diagnostic accuracy of doctors using MRCP combined with CT for the differentiation of malignant from benign causes of DBSs was higher than the diagnostic accuracy of MRCP alone. This result indicated that MRCP and enhanced CT have complementary roles; for example, thin-slice enhanced CT was better than MRCP in the observation of the bile duct wall, consistent with the report that combining different modalities is necessary to increase the diagnostic sensitivity (1).

In this study, we also found that stricture length, the angle of the DBS, and the presence of the double duct sign were significantly associated with malignant DBSs, while DBSs with low density in the arterial phase were negatively correlated with malignant DBSs.

Longer strictures were more commonly found in malignant DBSs than in benign DBSs. One reason may be that most of cholangiocarcinomas are infiltrative, especially distal cholangiocarcinomas, because they spread intramurally beneath the epithelium and along the wall of the bile duct (7, 11, 18). Another reason may be that abundant fibrosis due to the desmoplastic reaction in cholangiocarcinomas leads to bile duct wall thickening without mass formation but involving a long duct segment (6, 11). Kim et al. (6) showed that strictures with a length over 12 mm are more likely to be malignant, which is close to our result (9.5 mm). Some studies have also reported that narrowing of the long segment is associated with malignant strictures, but an optimal cutoff value is not easy to reach (7, 8, 11, 18). The reason may be that the extrahepatic bile duct distal to the stricture may be collapsed, potentially leading to an overestimation of the length of the stricture (12, 18).

In this study, malignant strictures usually manifested with irregular, abrupt narrowing with an obtuse angle, whereas benign strictures tended to have a smooth outer margin and gradual tapering with an acute angle. However, these factors exhibited no relationship upon multiple analyses, which is consistent with previous reports that abrupt or gradual tapering of strictures did not show a correlation with benign or malignant strictures (6, 7, 18). The reason may be that the angle of the DBS was introduced into this study. The angle of the DBS was measured quantitatively and was associated with the stricture pattern and the angle of the narrowing end, and this factor may lead to irregular, abrupt narrowing and the obtuse angle not being significant upon multiple analyses. We found that an angle of DBS larger than 84.1° was more likely to be a malignant DBS. Of course, these results still need to be confirmed.

The appearance of the “double duct sign”, which includes the dilation of the common bile duct and main pancreatic duct, is usually suggestive of a pancreatic head carcinoma. Although highly suggestive, other malignancies, such as cholangiocarcinoma and duodenal carcinoma, and benign entities, such as chronic pancreatitis, can also lead to the double duct sign (12). Because patients with chronic pancreatitis were excluded from this study, the double duct sign in MRCP and CT in the absence of an identifiable mass and chronic pancreatitis should suggest that DBS is likely to be malignant.

Hyperenhancement of a malignant stricture segment of the bile duct relative to the liver parenchyma was shown by previous studies. Choi et al. (8) showed that hyperenhancement of the involved stricture during the portal venous phase is the main factor distinguishing malignant strictures from benign strictures. Another study reported stronger enhancement of malignant bile duct strictures in the arterial and portal venous phases of CT (25). A similar result was described by Kim et al. (6), who showed that hyperenhancement was a significant factor for malignant strictures. In addition, Yu et al. (11) indicated that hyperenhancement during the equilibrium phase is more likely to indicate malignant strictures. The reason for hyperenhancement is associated with the relatively abundant vascular supply (11, 25). In this study, hyperenhancement was not related to malignant DBSs; the reason may be that inflammation can also lead to fibrosis, with significant enhancement in the venous and equilibrium phases. In contrast, we found that low density in the arterial phase was negatively correlated with malignant DBSs. This result does not conflict with the conclusion drawn from previous studies that hyperenhancement was related to malignant DBSs.

A scoring model based on the four factors was established for predicting the malignant risk of DBSs. When the cutoff value was 1.75, the sensitivity and specificity values for the malignancy of DBSs were 73.5 and 85.9%, respectively, and the positive and negative predictive values were 83.6 and 76.8%, respectively. However, there were some malignant DBSs with cutoff values lower than 1.75 and a few benign DBS scores higher than 1.75. One reason is that some patients’ slow blood circulation leads to lesion-delayed enhancement; another reason is that the stricture length may be overestimated due to a collapsed bile duct. The effectiveness of this scoring model needs to be verified by a prospective study.

### Limitations

Our study has several limitations. First, it was a retrospective nonrandomized study, and patients with cholangiolithiasis or non-DBS lesions were excluded; therefore, the effects of selection bias must be considered. Second, some DBSs defined as benign were confirmed by follow-up but not confirmed by pathology, and a few of these lesions might have become malignant in the future. Third, accurate measurement of stricture length, wall thickness, and the angle of the DBS was difficult in some patients. Fourth, this study considered only DBSs, and the results may not be applicable to other sites of biliary strictures. Fifth, although diffusion-weighted imaging (DWI) can help radiologists characterize bile duct lesions and detect extra-bile duct lesions (26), DWI was not used in this study because some patients had not undergone this imaging, or the image quality was poor. Sixth, compared to MRCP alone, contrast-enhanced CT brings additional contrast agent dose and x-ray exposure. However, the diagnostic accuracy of MRCP alone in differentiating malignant DBSs from benign DBSs is limited, especially for less experienced reviewers. When the diagnosis is not clear after MRCP, the invasive modalities like endoscopic diagnostic were performed and may lead to significant complications, whereas CT is noninvasive and faster. In addition, with the development of CT technology, exposure to radiation is gradually reduced. Further prospective studies with a large sample size are needed in the future. In addition, radiomics and deep learning may be helpful in identifying malignant and benign DBSs, and these are currently under investigation in our group.

## Conclusions

On the one hand, we found that MRCP combined with CT can increase the diagnostic accuracy for DBSs. On the other hand, we found that stricture length, the angle of the DBS, the presence of the double duct sign and low density in the arterial phase were correlated with malignant DBSs. The predictive scoring model based on the four factors is of great value in predicting the malignancy of DBSs. In clinical practice, radiologists should pay more attention to patients with DBSs when the score is ≥1.75.

## Data Availability Statement

The raw data supporting the conclusions of this article will be made available by the authors, without undue reservation.

## Ethics Statement

This retrospective study was approved by the institutional ethics committee of Xinqiao hospital (202013001). Written informed consent for participation was not required for this study in accordance with the national legislation and the institutional requirements.

## Author Contributions

LW: conceptualization. X-dG and DZ: data curation. H-lC and Q-cZ: investigation. LW: methodology. G-xW: writing—original draft. All authors contributed to the article and approved the submitted version.

## Funding

This study was supported by the Miaopu Foundation of Third Military Medical University (2019R064).

## Conflict of Interest

The authors declare that the research was conducted in the absence of any commercial or financial relationships that could be construed as a potential conflict of interest.

## Publisher’s Note

All claims expressed in this article are solely those of the authors and do not necessarily represent those of their affiliated organizations, or those of the publisher, the editors and the reviewers. Any product that may be evaluated in this article, or claim that may be made by its manufacturer, is not guaranteed or endorsed by the publisher.

## References

[1] Lee HJ Cho KB. Diagnosis of Malignant Biliary Stricture: More Is Better. Clin Endosc (2018) 51(2):115–7. doi: 10.5946/ce.2018.035 PMC5903087 29618174

[2] Bowlus CL Olson KA Gershwin ME. Evaluation of Indeterminate Biliary Strictures. Nat Rev Gastroenterol Hepatol (2016) 13(1):28–37. doi: 10.1038/nrgastro.2015.182 26526122

[3] Fernandez Y Viesca M Arvanitakis M. Early Diagnosis and Management of Malignant Distal Biliary Obstruction: A Review On Current Recommendations and Guidelines. Clin Exp Gastroenterol (2019) 12:415–32. doi: 10.2147/CEG.S195714 PMC6842280 31807048

[4] Xie C Aloreidi K Patel B Ridgway T Thambi-Pillai T Timmerman G. Indeterminate Biliary Strictures: A Simplified Approach. Expert Rev Gastroenterol Hepatol (2018) 12(2):189–99. doi: 10.1080/17474124.2018.1391090 29034764

[5] Xu MM Sethi A. Diagnosing Biliary Malignancy. Gastrointest Endosc Clin N Am (2015) 25(4):677–90. doi: 10.1016/j.giec.2015.06.011 26431597

[6] Kim JY Lee JM Han JK Kim SH Lee JY Choi JY. Contrast-Enhanced MRI Combined With MR Cholangiopancreatography for the Evaluation of Patients With Biliary Strictures: Differentiation of Malignant From Benign Bile Duct Strictures. J Magn Reson Imaging (2007) 26(2):304–12. doi: 10.1002/jmri.20973 17623893

[7] Park MS Kim TK Kim KW Park SW Lee JK Kim JS. Differentiation of Extrahepatic Bile Duct Cholangiocarcinoma From Benign Stricture: Findings at MRCP Versus ERCP. Radiology (2004) 233(1):234–40. doi: 10.1148/radiol.2331031446 15333766

[8] Choi SH Han JK Lee JM Lee KH Kim SH Lee JY. Differentiating Malignant From Benign Common Bile Duct Stricture With Multiphasic Helical CT. Radiology (2005) 236(1):178–83. doi: 10.1148/radiol.2361040792 15955859

[9] Liu X Yang Z Tan H Shao C Liu L Si S. Differentiation of Benign and Malignant Hilar Bile Duct Stenosis. J Surg Res (2016) 203(2):275–82. doi: 10.1016/j.jss.2016.03.002 27363632

[10] Chu YL Wang XF Gao XZ Qiao XL Liu F Yu SY. Endoscopic Ultrasonography in Tandem With Endoscopic Retrograde Cholangiopancreatography in the Management of Suspected Distal Obstructive Jaundice. Eur J Gastroenterol Hepatol (2013) 25(4):455–9. doi: 10.1097/MEG.0b013e32835ca1d7 23249605

[11] Yu XR Huang WY Zhang BY Li HQ Geng DY. Differentiation of Infiltrative Cholangiocarcinoma From Benign Common Bile Duct Stricture Using Three-Dimensional Dynamic Contrast-Enhanced MRI With MRCP. Clin Radiol (2014) 69(6):567–73. doi: 10.1016/j.crad.2014.01.001 24581958

[12] Katabathina VS Dasyam AK Dasyam N Hosseinzadeh K. Adult Bile Duct Strictures: Role of MR Imaging and MR Cholangiopancreatography in Characterization. Radiographics (2014) 34(3):565–86. doi: 10.1148/rg.343125211 24819781

[13] Nakai Y Isayama H Wang HP Rerknimitr R Khor C Yasuda I. International Consensus Statements for Endoscopic Management of Distal Biliary Stricture. J Gastroenterol Hepatol (2020) 35(6):967–79. doi: 10.1111/jgh.14955 PMC7318125 31802537

[14] Meeralam Y Al-Shammari K Yaghoobi M. Diagnostic Accuracy of EUS Compared With MRCP in Detecting Choledocholithiasis: A Meta-Analysis of Diagnostic Test Accuracy in Head-to-Head Studies. Gastrointest Endosc (2017) 86(6):986–93. doi: 10.1016/j.gie.2017.06.009 28645544

[15] Dorrell R Pawa S Zhou Y Lalwani N Pawa R. The Diagnostic Dilemma of Malignant Biliary Strictures. Diagnostics (Basel) (2020) 10(5):337. doi: 10.3390/diagnostics10050337 PMC7277979 32466095

[16] Singh A Mann HS Thukral CL Singh NR. Diagnostic Accuracy of MRCP as Compared to Ultrasound/CT in Patients With Obstructive Jaundice. J Clin Diagn Res (2014) 8(3):103–7. doi: 10.7860/JCDR/2014/8149.4120 PMC4003596 24783094

[17] Hanif H Khan SA Muneer S Adil SO. Diagnostic Accuracy of Ultrasound in Evaluation of Obstructive Jaundice With MRCP as Gold Standard. Pak J Med Sci (2020) 36(4):652–6. doi: 10.12669/pjms.36.4.1665 PMC7260912 32494250

[18] Suthar M Purohit S Bhargav V Goyal P. Role of MRCP in Differentiation of Benign and Malignant Causes of Biliary Obstruction. J Clin Diagn Res (2015) 9(11):TC08–12. doi: 10.7860/JCDR/2015/14174.6771 PMC4668504 26675498

[19] Romagnuolo J Bardou M Rahme E Joseph L Reinhold C Barkun AN. Magnetic Resonance Cholangiopancreatography: A Meta-Analysis of Test Performance in Suspected Biliary Disease. Ann Intern Med (2003) 139:547–57. doi: 10.7326/0003-4819-139-7-200310070-00006 14530225

[20] Batur A Kerimoglu U Ataseven H. Hounsfield Unit Density in the Characterisation of Bile Duct Lesions. Pol J Radiol (2019) 84:e397–401. doi: 10.5114/pjr.2019.89390 PMC6964322 31969956

[21] Chang S Lim JH Choi D Kim SK Lee WJ. Differentiation of Ampullary Tumor From Benign Papillary Stricture by Thin-Section Multidetector CT. Abdom Imaging (2008) 33(4):457–62. doi: 10.1007/s00261-007-9295-0 17712590

[22] Singh A Gelrud A Agarwal B. Biliary Strictures: Diagnostic Considerations and Approach. Gastroenterol Rep (2015) 3(1):22–31. doi: 10.1093/gastro/gou072 PMC4324869 25355800

[23] Kapoor BS Mauri G Lorenz JM. Management of Biliary Strictures: State-of-the-Art Review. Radiology (2018) 289(3):590–603. doi: 10.1148/radiol.2018172424 30351249

[24] Xie C Aloreidi K Patel B Ridgway T Thambi-Pillai T Timmerman G. Indeterminate Biliary Strictures: A Simplified Approach. Expert Rev Gastroenterol Hepatol (2018) 12(2):189–99. doi: 10.1080/17474124.2018.1391090 29034764

[25] Kim NR Lee JM Kim SH An SK Han CJ Choi SH. Enhancement Characteristics of Cholangiocarcinomas on Multiphasic Helical CT: Emphasis on Morphologic Subtypes. Clin Imaging (2008) 32(2):114e20. doi: 10.1016/j.clinimag.2007.08.022 18313575

[26] Lee DH Kim B Lee ES Kim HJ Min JH Lee JM. Radiologic Evaluation and Structured Reporting Form for Extrahepatic Bile Duct Cancer: 2019 Consensus Recommendations From the Korean Society of Abdominal Radiology. Korean J Radiol (2021) 22(1):41–62. doi: 10.3348/kjr.2019.0803 32901457 PMC7772383

